# Dimethyl sulfoxide’s impact on epileptiform activity in a mouse model of chronic temporal lobe epilepsy

**DOI:** 10.1016/j.eplepsyres.2023.107235

**Published:** 2023-09-30

**Authors:** Melanie Widmann, Andreas Lieb, Anna Mutti, Christoph Schwarzer

**Affiliations:** Department of Pharmacology, Medical University of Innsbruck, Innsbruck, Austria

**Keywords:** Dimethyl sulfoxide, DMSO, Intrahippocampal kainic acid model, Temporal lobe epilepsy, Pentylenetetrazol-induced acute seizures

## Abstract

In the quest for novel treatments for patients with drug-resistant seizures, poor water solubility of potential drug candidates is a frequent obstacle. Literature indicated that the highly efficient solvent dimethyl sulfoxide (DMSO) may have a confounding influence in epilepsy research, reporting both pro- and antiepileptic effects. In this study, we aim to clarify the effects of DMSO on epileptiform activity in one of the most frequently studied models of chronic epilepsy, the intrahippocampal kainic acid (IHKA) mouse model, and in a model of acute seizures. We show that 100 % DMSO (in a volume of 1.5 μl/g corresponding to 1651 mg/kg) causes a significant short-term anti-seizure effect in epileptic IHKA mice of both sexes, but does not affect the threshold of acute seizures induced by pentylenetetrazol (PTZ). These findings highlight that the choice of solvent and appropriate vehicle control is crucial to minimize undesirable misleading effects and that drug candidates exclusively soluble in 100 % DMSO need to be modified for better solubility already at initial testing.

## 1 Introduction

Epilepsies rank among the most prevalent neurological diseases worldwide. The fact that more than one-third of patients have inadequate seizure control with currently available anti-seizure medications (Chen et al., 2018) highlights the urgent need to develop novel treatments. In this quest poor water solubility of potential drug candidates is a frequent obstacle.

Dimethyl sulfoxide (DMSO) is one of the most efficient solvents for water-insoluble compounds (Santos et al., 2003), however, its application in epilepsy research may be problematic due to potential effects on neuronal excitability. Seizure development and neurotoxicity were reported in studies using DMSO as cryoprotectant in stem cells transfusions (Ataseven et al., 2017; Bauwens et al., 2005; Maral et al., 2018). Existing data are inconsistent, reporting both pro- (Kumari et al., 2018; Tamagnini et al., 2014; Kovács et al., 2011) and antiepileptic effects (Tamagnini et al., 2014; Kovács et al., 2011; Maclennan et al., 1996; Larsen et al., 1996; Carletti et al., 2013) depending on the experimental model and concentration of DMSO. To our knowledge, DMSO has not yet been investigated in the intrahippocampal kainic acid (IHKA) mouse model of chronic temporal lobe epilepsy (TLE), which is incorporated into the current epilepsy therapy screening program of the NIH (Kehne et al., 2017).

## 2 Materials and methods

### 2.1 Animals

Animal experiments were designed according to ARRIVE guidelines and the Basel declaration and approved by the Austrian Animal Testing Commission in compliance with the EU Directive 2010/63/EU.

C57BL/6 N mice (8–10 weeks at surgery) were housed in type II-L individually ventilated cages under standard conditions (12 h light-dark cycle, lights on 7 am–7 pm, 22 ± 2 °C, 45–65 % relative humidity, freely accessible food and water) in groups of 5 before and single-caged after surgery.

### 2.2 Kainic acid injection and electrode implantation

11 male and 12 female mice underwent stereotaxic IHKA injection and implantation of local field potential (LFP) electrodes as previously described (Zangrandi et al., 2016). In brief, a 50 nl bolus of 20 mM kainic acid (Ocean Produce International) was injected into the area CA1 of the left hippocampus (AP –1.8 mm, ML +1.2 mm, DV –1.8 mm to bregma). Depth electrodes were implanted bilaterally into CA1 (AP –1.8, ML ± 1.3, DV –1.8), a surface electrode above the motor cortex and reference electrodes above the cerebellum. Status epilepticus was confirmed in all mice by recordings immediately after surgery. 2 male mice died during status epilepticus.

### 2.3 Experimental treatments, LFP recordings and analysis

8 male and 9 female mice with established chronic epilepsy (meeting the inclusion criterium of *>*50 s/h HPDs; 1 male and 3 females did not develop sufficient HPDs) were treated s.c. with saline 0.9 % or fresh DMSO (1 %, 10 %, 30 % and 100 % in a volume of 1.5 μl/g corresponding to 16.5 mg/kg, 165.1 mg/kg, 495.2 mg/kg and 1651 mg/kg) in a Latin square design. The minimum resting period between injections was 72 h.

LFPs were acquired from freely moving animals using wireless Neurologgers (TSE systems). LFP signals, filtered between 0.5 and 70 Hz using a second-order Butterworth bandpass filter, were divided into 1 s long intervals of which the minimum or maximum (depending on epileptic spike polarity) was determined and the mode calculated. Spike trains were defined as series of ≥3 epileptiform spikes (amplitude 2x mode, minimum distance between spikes 70 ms) lasting 1–10 s with ≥1.33 Hz. Trains of ≥10 s were counted as hippocampal paroxysmal discharges (HPDs). 5 min before and after the injection were excluded to minimize the influence of handling. Recordings with <100 s spike trains and <50 s HPDs during the 1 h pre-treatment or a generalized seizure during the analyzed period were excluded.

Using the Fast Fourier transform (FFT) algorithm with a 10 s sliding Hanning window the LFP signal power was calculated for 1–4 Hz (delta), 4–8 Hz (theta), 8–13 Hz (alpha), 13–30 Hz (beta) and 30–80 Hz (gamma) frequency bands, typically used for rodent studies (Kadam et al., 2017). In addition, the coastline, an aggregate of frequency and amplitude, was computed as sum of the absolute difference between successive points (Lieb et al., 2018).

### 2.4 Pentylenetetrazol seizure threshold

We determined the threshold for PTZ-induced acute seizures after pretreatment (25 ± 1 min before start of PTZ infusion in accordance with the maximum effect of DMSO in the IHKA model, see Supplemental Fig. A.1) with different concentrations of DMSO and controls. PTZ (10 mg/ML in 0.9 % saline, Merck KGaA) was infused at 100 μl/min into the tail vein of freely moving animals and stopped upon onset of tonic-clonic seizures. The seizure threshold was calculated from the infused volume relative to the body weight as previously described (Loacker et al., 2007).

### 2.5 Data and statistical analysis

Statistical analyses were performed with GraphPad Prism 9.5.1. A two-way mixed-effects model for repeated measures followed by Dunnett’s multiple comparisons test (in comparison to saline control) was applied for data from IHKA mice after treatment with different DMSO concentrations normalized to the pre-treatment baseline. The absolute pre- and post-treatment data were analyzed with a three-way mixed-effects model for repeated measures followed by Šidák’s multiple comparisons test. PTZ seizure threshold data were analyzed using ordinary two-way ANOVA. P-values <0.05 were considered statistically significant. Data are presented as mean ± standard deviation (SD), with data for individual animals shown.

## 3 Results

### 3.1 Effect of DMSO in the IHKA model of TLE

**Fig. 1:**
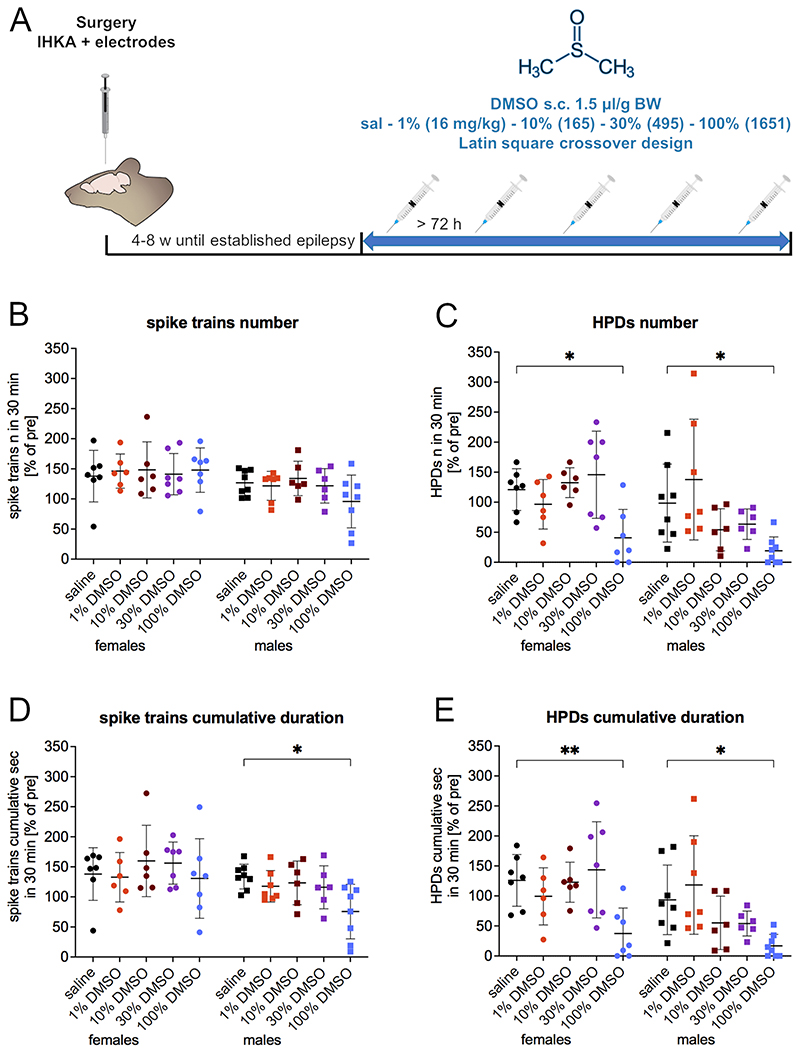
Effect of different concentrations of DMSO on spike trains and HPDs in the IHKA mouse model of TLE. (A) Experimental timeline for testing of different concentrations of DMSO (1 %, 10 %, 30 % and 100 % in a volume of 1.5 μl/g corresponding to 16.5 mg/kg, 165.1 mg/kg, 495.2 mg/kg and 1651 mg/kg) vs. 0.9 % saline (sal) s.c. in mice with established chronic epilepsy 4–8 weeks after IHKA. Treatment schedules were designed according to a Latin square crossover design with 72 h minimum resting period between injections. (B) Number [% of pre] and (D) cumulative duration [% of pre] of spike trains and (C) number [% of pre] and (E) cumulative duration [% of pre] of HPDs in female and male IHKA mice in the 5–34 min after treatment with 1 %, 10 %, 30 % and 100 % DMSO compared to saline. Regarding HPDs, number and cumulative duration were significantly reduced after 100 % DMSO in both females and males in comparison to saline. 100 % DMSO in male mice resulted in a significant reduction of the cumulative duration of spike trains compared to saline. Data (females n = 9, males n = 8) are presented as % normalized to the pretreatment period (mean ± SD) and were analyzed with a 2-way linear mixed model for repeated measures followed by Dunnett’s multiple comparisons test. * p < 0.05; * * p < 0.01.

The effect of DMSO on epileptiform activity was evaluated in the IHKA model of TLE. In female and male mice with established chronic epilepsy after IHKA, we tested concentrations of DMSO ranging from 1 % to 100 % vs. saline (Fig. 1A). A short-lasting decrease of epileptiform activity was observed after 100 % DMSO (in a volume of 1.5 μl/g corresponding to 1651 mg/kg) compared to saline (Supplemental Fig. A.1). Number and cumulative duration of spike trains and HPDs were evaluated 5–34 min after treatment and normalized to the last hour before the treatment. Treatment with 100 % DMSO resulted in a significant reduction of HPDs (Fig. 1C and E) in females and males: number (females 40.8 ± 47.1 %; 95 % CI 12.31–147.9, p = 0.0159; males 19.2 ± 23.1 %; 95 % CI 15.79–142.9, p = 0.0100) and cumulative duration (females 37.3 ± 42.5 %; 95 % CI 21.46–155.9, p = 0.0062; males 16.9 ± 19.0 %; 95 % CI 13.63–139.6, p = 0.0125) in comparison to saline (number: females 120.9 ± 34.8 %, males 98.5 ± 64.9 %; cumulative duration: females 126.0 ± 43 %; males 93.6 ± 58.0 %). The effect on spike trains was less pronounced, the cumulative duration (Fig. 1D) showed a significant reduction only in male mice after injection of 100 % DMSO (75.6 ± 45.3 %, 95 % CI 7.767–108.8, p = 0.0190) compared to saline (133.9 ± 20.7 %), while the number of spike trains was not affected (Fig. 1B). Intriguingly, the two-way mixed-effects model for repeated measures indicated significant sex-specific differences for all analyzed parameters (spike trains number: p = 0.0456, F (1, 15) = 4.752; cumulative duration: p = 0.0414, F (1, 15) = 4.973; HPDs number: p = 0.0463, F (1, 15) = 4.717; cumulative duration: p = 0.0156, F (1, 15) = 7.440). We did not observe any significant rebound increase during a later time interval of 35–94 min (Supplemental Fig. A.2).

The results of the normalized data were further corroborated by analyzing the absolute pre- and post-treatment data (Supplemental Fig. A.3). HPDs were significantly reduced after 100 % DMSO in females (number: 3.0 ± 3.8 n/h, 95 % CI 0.7–12.2, p = 0.0205; cumulative duration: 46.8 ± 62.2 s/h, 95 % CI 19.1–216.1, p = 0.0115) and males (number: 1.4 ± 1.8 n/h, 95 % CI 2.4–13.1, p = 0.0016; cumulative duration: 18.9 ± 22.8 s/h, 95 % CI 29.5–213.8, p = 0.0042) compared to pre-treatment (females number 9.4 ± 5.2 n/h and cumulative duration 164.4 ± 100.3 s/h; males number 9.1 ± 4.5 n/h and cumulative duration 140.5 ± 74.6 s/h), while saline did not show statistically significant effects. The number of spike trains appeared significantly increased in female mice after 10 % (80.2 ± 31.9 n/h, 95 % CI −48.8 to −0.4, p = 0.0441) and 100 % DMSO (85.6 ± 43.0 n/h, 95 % CI −51.9 to −7.1, p = 0.0043) compared to pre-treatment (10 % DMSO: 55.6 ± 21.7 n/h 100 % DMSO: 56.1 ± 18.5 n/h).

**Fig. 2:**
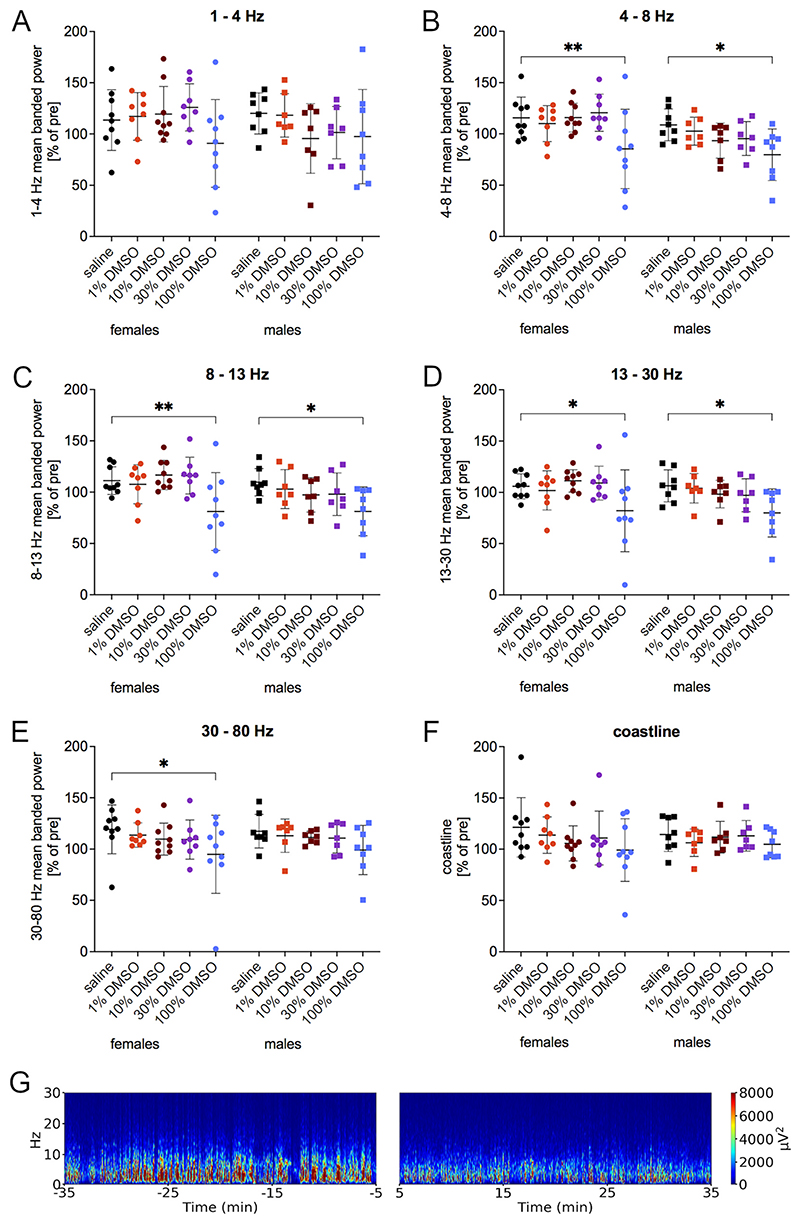
Banded power after different concentrations of DMSO in the IHKA mouse model of TLE. Power in the (A) 1–4 Hz, (B) 4–8 Hz, (C) 8–13 Hz, (D) 13–30 Hz and (E) 30–80 Hz frequency bands as well as the (F) coastline were calculated for the 5–34 min after treatment normalized to the pretreatment period. 100 % DMSO caused a significant decrease in the 4–8 Hz, 8–13 Hz and 13–30 Hz bands in both sexes and in the females additionally in the 30–80 Hz band. Data (females n = 9, males n = 8) are presented as % normalized to the pretreatment period (mean ± ŞD) and were analyzed with a 2-way linear mixed model for repeated measures followed by Dunnett’s multiple comparisons test. * p=<0.05; ** p=<0.01. (G) Representative spectrogram before and after 100 % DMSO showing a reduction.

Treatment with 100 % DMSO resulted in significant alterations of the power spectrum (Fig. 2G). 5–34 min after 100 % DMSO, power in the 4–8 Hz (Fig. 2B; females 85.4 ± 38.6 %, 95 % CI 6.422–54.21, p = 0.0084; males 79.7 ± 25.1 %, 95 % CI 3.793–54.53, p = 0.0192), 8–13 Hz (Fig. 2C; females 81.1 ± 37.9 %, 95 % CI 7.220–53.17, p = 0.0061; males 81.3 ± 23.7 %, 95 % CI 3.952–52.73, p = 0.0177) and 13–30 Hz bands (Fig. 2D; females 82.0 ± 39.9, 95 % CI 1.601–46.05, p = 0.0320; males 79.9 ± 23.5, 95 % CI 2.884–50.07, p = 0.0231) was significantly decreased in both sexes (saline: 4–8 Hz: females 115.7 ± 20.2, males 108.9 ± 15.5; 8–13 Hz: females 111.3 ± 13.5, males 109.7 ± 13.3; 13–30 Hz: females 105.8 ± 12.2, males 106.4 ± 15.6) and in females additionally in the 30–80 Hz band (Fig. 2E; 94.9 ± 38.1, 95 % CI 3.376–45.25, p = 0.0178; saline: 119.2 ± 23.8). DMSO did not affect power in the 1–4 Hz band (Fig. 2A) and the coastline (Fig. 2F).

### 3.2 Effect of DMSO on acute seizures

**Fig. 3:**
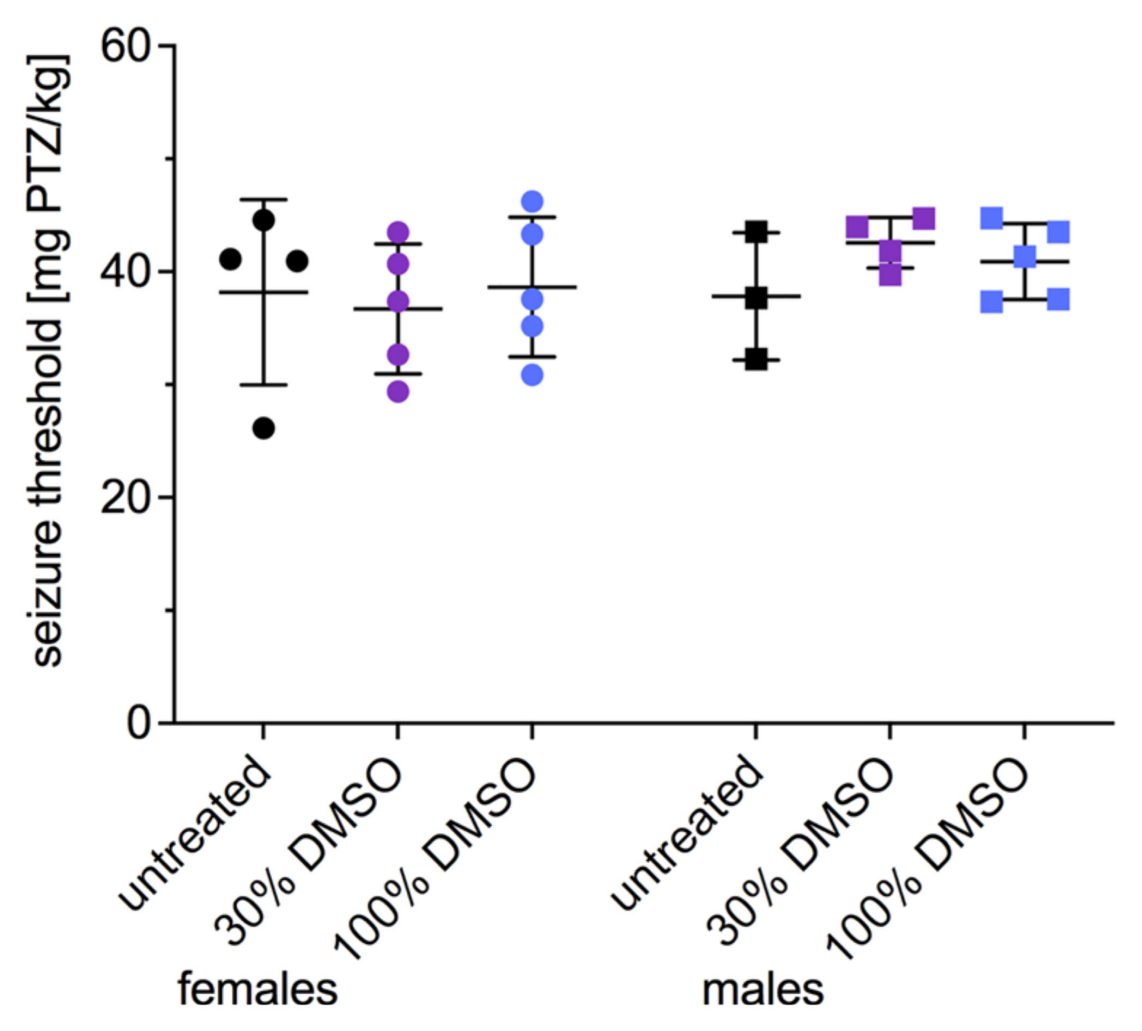
PTZ-induced acute seizure threshold after different concentrations of DMSO. Seizure threshold [mg PTZ/kg] determined by PTZ tail-vein infusion in untreated animals or pretreated with 30 % or 100 % DMSO showed no statistically significant differences. Data (n = 3–5) are expressed as mean ± SD and were analyzed with ordinary 2-way ANOVA.

To evaluate whether DMSO influences acute seizures in non-epileptic mice, we determined the seizure threshold by infusing PTZ via the tail vein in untreated mice or pretreated with 30 % or 100 % DMSO (Fig. 3). No significant differences were observed.

## 4 Discussion

In drug development, poor water solubility of drug candidates is a frequent obstacle. Since literature indicated that the highly efficient solvent DMSO may have confounding influence in epilepsy research (Ataseven et al., 2017; Bauwens et al., 2005; Maral et al., 2018; Kumari et al., 2018; Tamagnini et al., 2014; Kovács et al., 2011; Maclennan et al., 1996; Larsen et al., 1996; Carletti et al., 2013), we investigated its effects on epileptiform activity in IHKA mice. Our results suggest that DMSO concentrations below 30 % (in a volume of 1.5 μl/g corresponding to 495.2 mg/kg) minimize confounding effects. This is in accordance with a study in a rat model of electrically induced temporal lobe seizures showing reduced maximal dentate activation after a dose of 1651 mg/kg DMSO (Carletti et al., 2013). In a rat model of genetic absence epilepsy, the contrary effect of increased epileptic activity was observed during later time intervals starting 30 min after high doses of DMSO (825 mg/kg and 1651 mg/kg) (Kovács et al., 2011). We observed a rapid onset but short-lasting decrease of epileptiform activity after 1651 mg/kg DMSO and no significant rebound increase during the later time interval of 35–94 min after treatment.

Frequently, poorly water-soluble drug candidates are dissolved in pure DMSO as a stock solution and subsequently diluted with saline. However, it has to be taken into consideration that a solvent mixture such as 10 % DMSO and 90 % saline is administered with a higher volume of typically 10 μl/g body weight, reaching an absolute DMSO dose (1100 mg/kg) in a similar range as the 100 % DMSO 1.5 μl/g in our study (1651 mg/kg).

The potential mechanisms underlying DMSO-induced effects are not clearly understood so far. DMSO is known to affect cell membranes by interacting with the lipid constituents (Gurtovenko and Anwar, 2007). Furthermore, DMSO blocks the activation of sodium channels (Larsen et al., 1996), decreases NMDA receptor-, AMPA receptor- (Lu and Mattson, 2001) and GABA receptor-induced ion currents (Nakahiro et al., 1992), attenuates potassium currents and may change properties of T- and L-type calcium channels (Santos et al., 2003). Due to this influence on different ion channels, DMSO could shift the excitation/inhibition balance and thereby affect neuronal excitability. Accumulating evidence supports the involvement of impaired ion homeostasis, in particular chloride homeostasis in epilepsy (Fritschy, 2008). Thus, the interference of DMSO might be more pronounced in an epileptic system with preexisting disturbances compared to acute seizures in a healthy system.

In conclusion, we showed that the solvent 100 % DMSO (1.5 μl/g corresponding to 1651 mg/kg) caused a significant short-term reduction of HPDs in IHKA mice of both sexes to different extents, highlighting that the choice of solvent and appropriate vehicle control is crucial to minimize undesirable misleading effects. DMSO, due to its toxic effects, cannot be administered to humans and therefore drug candidates exclusively soluble in 100 % DMSO need to be modified for enhanced water solubility anyways. Our data show that such modifications are needed already for initial testing in epilepsy models.

## Supplementary Material

Supplementary material

## Data Availability

The data that support the findings of this study are available from the corresponding author upon reasonable request.
