# Supplementary material for: Dimethyl sulfoxide’s impact on epileptiform activity in a mouse model of chronic temporal lobe epilepsy
Source: Epilepsy Res. Author manuscript; Available in PMC 2023 Oct 25. (PMC7615238; doi:10.1016/j.eplepsyres.2023.107235)
Supplement: Supplementary material [file EMS189417-supplement-Supplementary_material.docx]

APPENDIX A


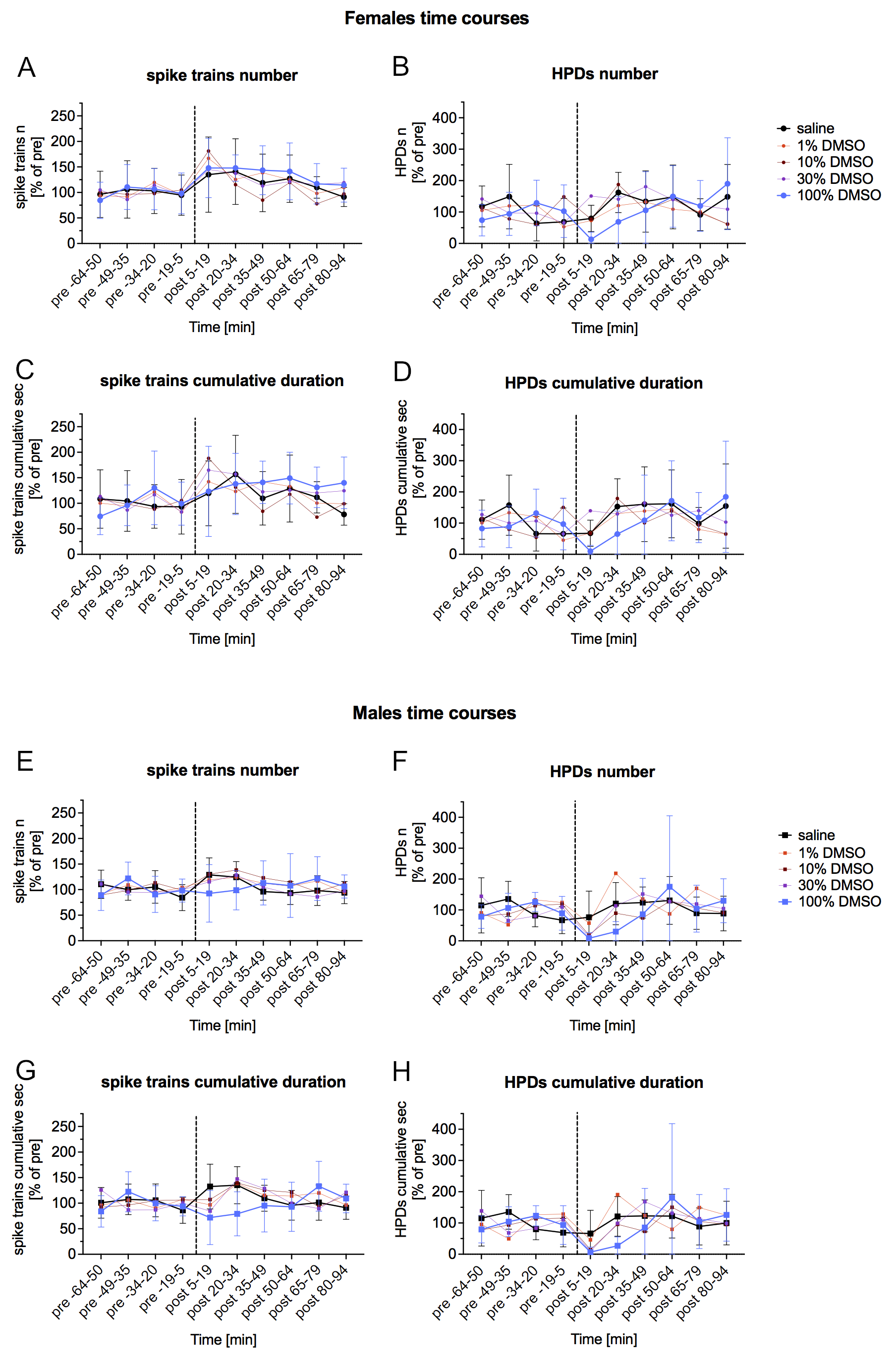


*Supplemental Figure A.1.* Time courses showing the effect of different concentrations of DMSO in female (A-D) and male (E-H) IHKA mice.

(A, E) Number [% of pre] and (C, G) cumulative duration [% of pre] of spike trains and (B, F) number and (D, H) cumulative duration of HPDs are depicted. Data (mean ± SD for saline and 100 % DMSO; for reasons of clarity only mean for 1 %, 10 % and 30 % DMSO) are presented in 15 min bins as % normalized to an average of the pretreatment period for the 64-5 min before and 5-94 min after the treatment, excluding a handling period of 5 min before and after the treatment. Note the decrease of number and cumulative duration of HPDs in the 5-19 and 20-34 min bins after 100 % DMSO compared to saline. Treatment time points are marked by a vertical dashed line. The saline and 100 % DMSO curves are highlighted with thicker lines.


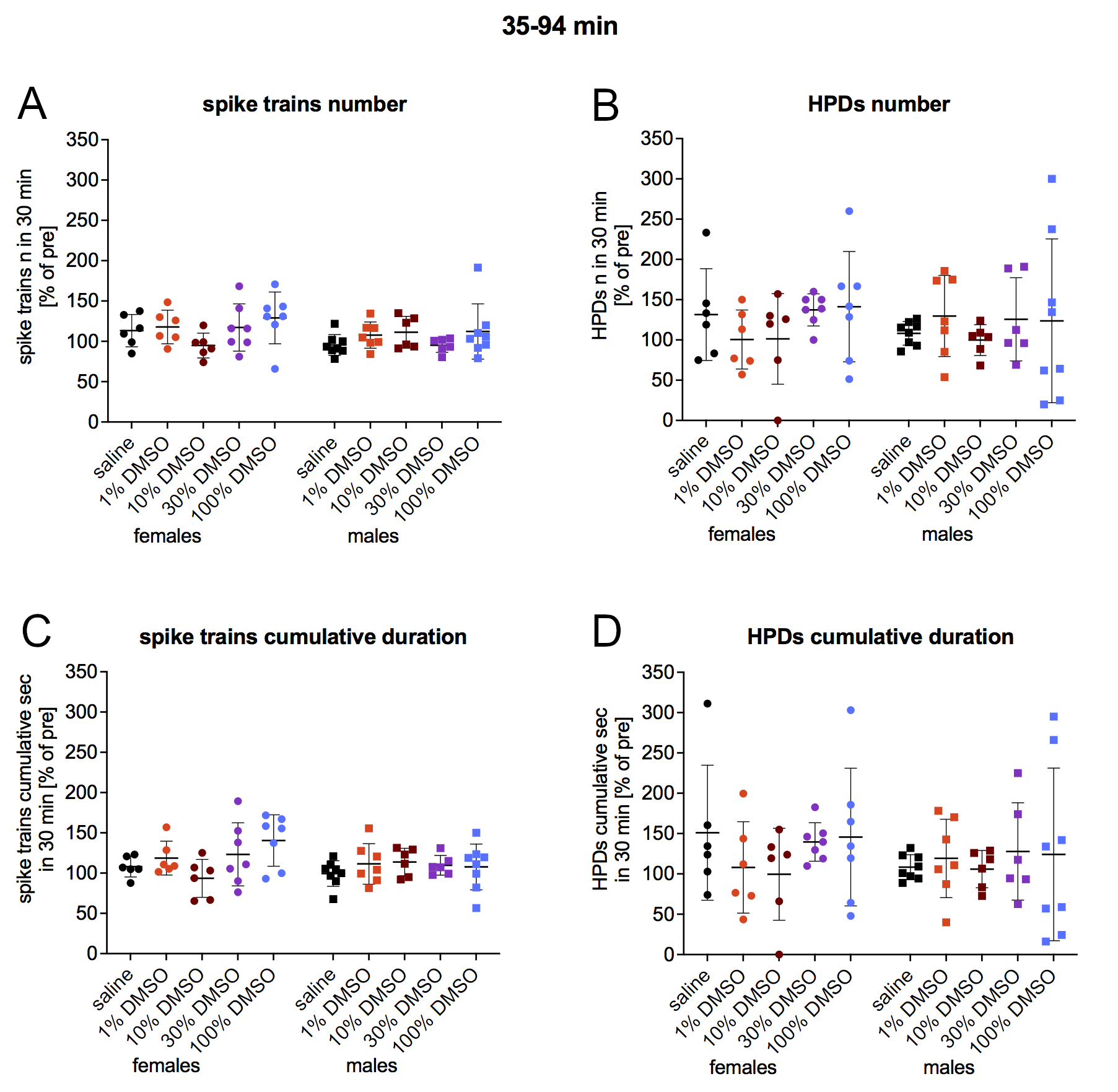


***Supplemental Figure A.2.* Analysis of the 35-94 min after treatment with different concentrations of DMSO in IHKA mice.**

(A) Number [% of pre] and (C) cumulative duration [% of pre] of spike trains and (B) number [% of pre] and (D) cumulative duration [% of pre] of HPDs in the 35­–94 min after 1 %, 10 %, 30 % and 100 % DMSO compared to saline in female and male IHKA mice are shown. No significant rebound increase was observed in the 35-94 min interval. Data (females n = 9, males n = 8) are presented as % normalized to the pretreatment period (mean ± SD) and were analyzed with a 2-way linear mixed model for repeated measures followed by Dunnett’s multiple comparisons test.


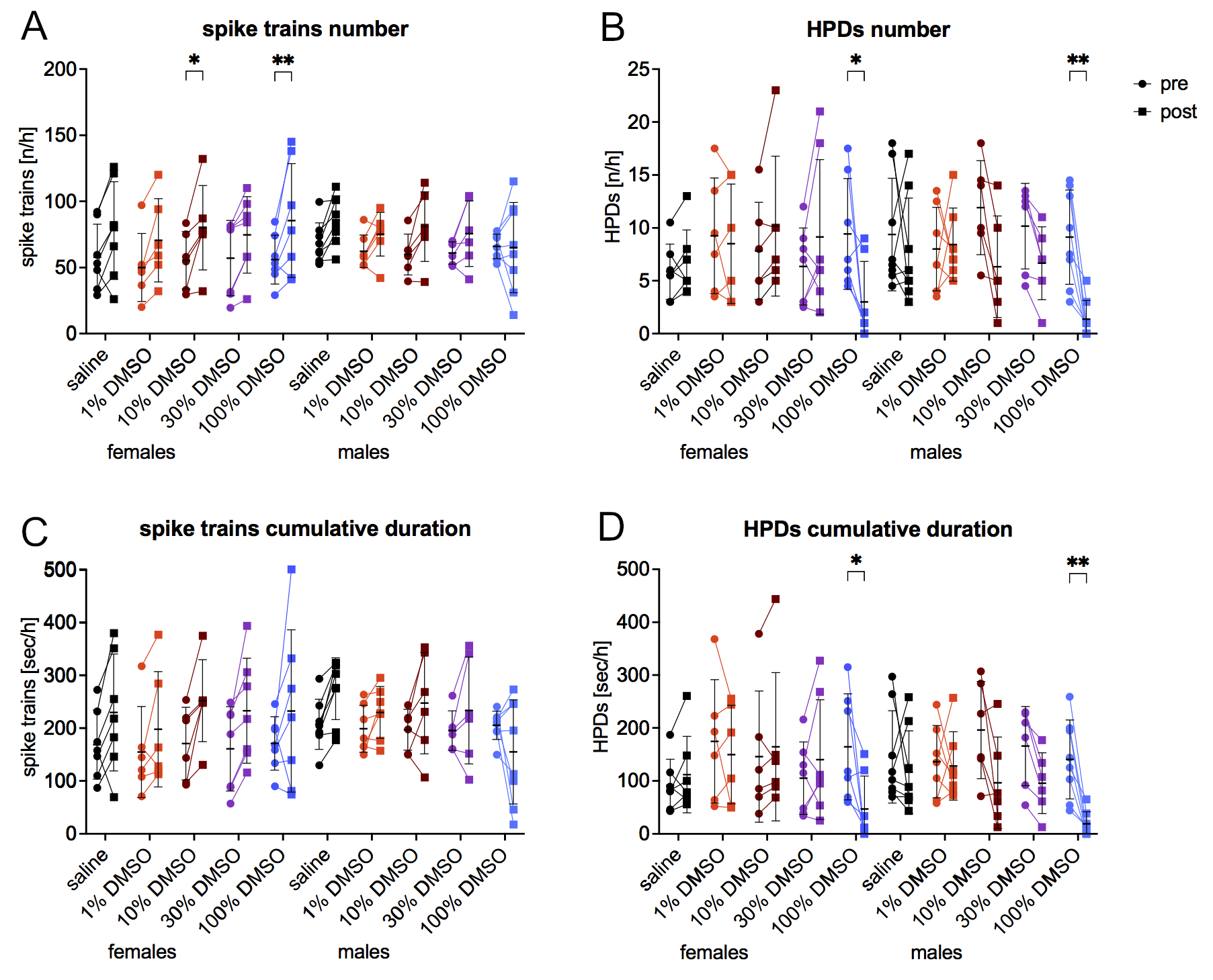


***Supplemental Figure A.3.* Analysis of spike trains and HPDs pre- and post-treatment with saline and different concentrations of DMSO in female and male IHKA mice.**

(A) Number [n/h] and (C) cumulative duration [sec/h] of spike trains and (B) number [n/h] and (D) cumulative duration [sec/h] of HPDs before and after saline, 1 %, 10 %, 30 % and 100 % DMSO in female and male IHKA mice are shown. HPDs were significantly reduced after 100 % DMSO in females and males. Female mice showed an increased number of spike trains after 10% and 100% DMSO. Data (females n = 9, males n = 8) were analyzed with a 3-way linear mixed model for repeated measures followed by Šidák’s multiple comparisons test and are presented as individual values with mean ± SD also shown. * p = < 0.05; ** p < 0.01
